# Obituary

**Published:** 1839

**Authors:** 


### OBITUARY.

Died, on the 28th of August, 1839, Mr. David Key, Surgeon, of St. John's, Southwark, in his 79th year, universally respected, having lived in that parish nearly 60 years.

